# Weekly chemotherapy as first line treatment in frail head and neck cancer patients in the immunotherapy era

**DOI:** 10.1186/s12967-021-02975-3

**Published:** 2021-07-12

**Authors:** Andrea Botticelli, Giulia Pomati, Alessio Cirillo, Giulia Mammone, Fabio Ciurluini, Bruna Cerbelli, Paolo Sciattella, Massimo Ralli, Umberto Romeo, Francesca De Felice, Carlo Catalano, Francesco Vullo, Marco Della Monaca, Sasan Amirhassankhani, Silverio Tomao, Valentino Valentini, Marco De Vincentiis, Vincenzo Tombolini, Carlo Della Rocca, Antonella Polimeni, Cira di Gioia, Alessandro Corsi, Giulia D’Amati, Silvia Mezi, Paolo Marchetti

**Affiliations:** 1 Department of Clinical and Molecular Oncology, “Sapienza” University of Rome, 00185 Rome, Italy; 2 Department of Molecular Medicine, “Sapienza” University of Rome, 00185 Rome, Italy; 3 Department of Radiological, Oncological and Anatomo-Pathological Science, “Sapienza” University of Rome, Viale Regina Elena 324, 00185 Rome, Italy; 4 Department of Statistical Sciences, Sapienza University of Rome, 00161 Rome, Italy; 5 Odontostomatological and Maxillo-Facial Science, ‘Sapienza’ University of Rome, 00185 Rome, Italy; 6 MSc Guy’s & St Thomas’ NHS Foundation Trust, Westminster Bridge Rd, Bishop’s, London, SE1 7EH UK; 7 Department of Medico-Surgical Sciences and Biotechnology, Sapienza University, Polo Pontino, 00185 Rome, Italy

**Keywords:** Head and neck cancer, Docetaxel, Cetuximab, Chemotherapy, First line, Frail patient population

## Abstract

**Objective:**

First-line therapy for metastatic squamous cell carcinoma of the head and neck (R/M HNSCC) has been revolutionized by the introduction of anti-checkpoint monoclonal antibodies, which have shown a significant improvement in overall survival (OS) gaining approval in a first line setting. Efficacy and safety of first-line weekly chemotherapy, compared to 3-weeks treatment, was retrospectively evaluated in a frail patient population with R/M HNSCC with the aim to evaluate its role as part of a personalized first-line approach.

**Methods:**

A total of 124 patients with locally incurable R/M HNSCC receiving weekly (21) or three-weekly (103) chemotherapy plus cetuximab in a first line setting from December 2010 to September 2020 were retrospectively reviewed. Treatment outcomes in terms of objective response rate (ORR), progression-free survival (PFS), overall survival (OS) and toxicities were analysed.

**Results:**

Patients in the three-week subgroup were ECOG PS 0 (39) and 1 (64) while patients in weekly group (21) were all PS 2. No significant differences were reported in terms of age, sex, smoking and previous alcohol abuse considering the two distinct subgroups. Moreover, no statistically significant difference was found in PFS and OS between the two treatment subgroups. The response rate was 35% (36 patients) and 34% (7 patients) in three-week and weekly treatment group, respectively. Seventy patients (68%) in the three-week group experienced chemotherapy-related toxicities, predominantly G3. In the weekly group a predominantly low-grade toxicity was found in a lower number of patients (52%).

**Conclusion:**

The weekly schedule appears to be an active and safe strategy in frail patients with R/M HNSCC. Based on these data, a weekly schedule could be considered as a first line treatment in all frail patients excluded from pembrolizumab treatment and a study on the combination of weekly chemotherapy and immunotherapy should be performed.

## Introduction

Head and neck squamous cell carcinoma (HNSCC) represents a heterogeneous spectrum of diseases originating predominantly from oral cavity, oropharynx, hypopharynx, and larynx [1]. HNSCC is globally the sixth most common type of cancer with 830,000 new cases and around 430,000 deaths each year worldwide [2]. Recurrent/metastatic head and neck squamous cell carcinoma (R/M-HNSCC), which is locally incurable, is associated with a poor prognosis [3. The old standard treatment for R/M HNSCC consisted of platinum-based chemotherapy plus cetuximab, according to the therapeutic regimen EXTREME [4]. In a phase III trial, Vermorken et al. demonstrated that adding cetuximab to platinum-5Fluoruracil (5FU) chemotherapy prolonged the median OS and PFS (from 7.4 months to 10.1 months and from 3.3 to 5.6 months, respectively). There were no significant differences in terms of Grade 3 and 4 toxicities between the two arms (82% and 76% in cetuximab and chemotherapy alone arm, respectively); however, in the cetuximab arm there were significantly more cases of sepsis (p = 0.02) and hypomagnesemia (0.05) compared to the platinum-based chemotherapy only one [4]*.*

Subsequently, a large randomized trial confirmed the good survival outcomes and response rate of the taxane-based TPE-x regimen observed in the phase II GORTEC study in first line R/M HNSCC [5]. Despite the lack of significant increase of overall survival (OS) when compared to EXTREME, TPEx required a shorter duration (4 vs 6 cycles) with a lower toxicity profile resulting in improvement of quality of life [6]. Furthermore, recent evidences have shown that docetaxel was able to act on the tumour microenvironment favouring the priming of immune response. This mechanism supports the hypothesis that taxanes act synergically with immunotherapy, highlighting the importance of their use in clinical practice [7–10].

A large proportion of patients with R/M HNSCC have poor clinical conditions, weight loss, signs and symptoms related to the extent of the cancer such as pain or obstruction, comorbidities and impaired organ function, therefore they are not suitable for EXTREME treatment due to the high risk of developing high grade toxicity. Conversely, a weekly chemotherapy regimen plus cetuximab was considered a reliable therapeutic strategy in frail cancer patients with R/M-HNSCC [11]. Weekly taxane-based chemotherapy has proved to control cancer growth and its related symptoms, with reduced toxicity and with an appropriate safety profile [12].

First-line therapy for R/M-HNSCC has been revolutionized by the introduction of immune-checkpoint monoclonal antibodies inhibitors (ICIs), a class of drugs targeting the inhibitory immune-checkpoint receptors. The results of the open label randomized phase 3 study KEYNOTE-048 led to approval of the anti PD-1 pembrolizumab in a first line setting, alone or in combination with cisplatin/5 fluorouracil-based chemotherapy [13]. The study evidenced a significantly prolonged OS vs. EXTREME regimen in patients with PD-L1 combined positive score (CPS) > / = 20 and CPS > / = 1. Toxicity profile was favourable for pembrolizumab vs. EXTREME and comparable vs. EXTREME for pembrolizumab plus chemotherapy [13].

Nevertheless, some issues are still to be tackled. The KEYNOTE-048 study enrolled patients in good general condition and with ECOG Performance Status (PS) = 0/1, although R/M HNSCC with poor clinical condition (PS = 2) and related signs/symptoms were excluded from this clinical changing study, making its results difficult to be extended to a population of frail patients; in this context the combination strategy still remains an unresolved issue.

This study retrospectively observed the clinical outcomes and toxicities of patients with poor baseline clinical condition treated in a first line setting with a weekly taxane-based chemotherapy, compared to patients with good PS, treated with an EXTREME like chemotherapy regimen.

## Materials and methods

### Patients

Data from patients with R/M HNSCC who received first line chemotherapy in association with cetuximab in our center from December 2010 to September 2020 were retrospectively analysed.

Patients were clinically staged with contrast enhanced computerized tomography (CT) scan and magnetic resonance imaging (MRI) before starting first line chemotherapy. All patients were discussed and judged as non-eligible for local/regional treatments by the multidisciplinary team of our hospital. Data including age, sex, ECOG PS, comorbidities, history of tobacco smoking and alcohol abuse, primary tumor sites, site of relapse (local/regional vs. metastatic) and chemotherapy treatment schedule were retrospectively collected. On the basis of PS, related symptoms, age, nutritional status and comorbidities, patients were judged as either frail or clinically fit and therefore scheduled for the two different treatments (EXTREME regimen or weekly chemotherapy).

Patients who had comorbidities contraindicating platinum-based chemotherapy and/or cetuximab were not included in the analysis, as well as all patients deemed unsuitable for chemotherapy who have undergone best supportive care.

### Treatment and assessments

Three weekly chemotherapy sessions, according to the EXTREME regimen of cisplatin at a dose of 100 mg/m^2^ of body-surface area on day 1, or carboplatin at an area under the curve (AUC) of 5 mg/ml/minute on day 1, plus fluorouracil (5-FU) at a dose of 1000 mg/m^2^/day for 4 days, plus cetuximab at a dose of 400 mg/m^2^ as a loading dose, followed by a dose of 250 mg/m^2^ per week every 21 days for a maximum of 6 cycles, were administered to patients considered fit at the baseline clinical evaluation. Cetuximab maintenance was performed in all patients who achieved at least stable disease (SD) as their best response.

Weekly chemotherapy according to the modified schedule of paclitaxel 80 mg/m^2^ on day 1,8,15, carboplatin AUC 2 on day 1, 8, 15 and cetuximab at a 400 mg loading dose followed by 250 mg/m^2^ weekly every 21 days (PCC), were administered intravenously to those patients deemed frail and unfit for the EXTREME regimen.

Tumor response was assessed every 12 weeks using Response Evaluation Criteria in Solid Tumors (RECIST) guidelines and classified as complete response (CR), partial response (PR), SD, and progressive disease (PD). Toxicities were recorded at day 1 of every cycle and classified according to the National Cancer Institute Common Terminology Criteria for Adverse Events (version 4.0). Progression-free survival (PFS) was defined as the time from the administration of treatment until the first progression or treatment death. The OS was defined as the time from patient registration to death from any cause.

## Statistical analysis

In the descriptive analysis quantitative variables were described as median and range, while qualitative variables were reported as number and percentage. The association between each clinical/pathological feature and outcome was evaluated using univariate and multivariable logistic regression models. Statistical significance was set at p < 0.05. All analyses were performed using SAS 9.4 (SAS Institute Inc., Cary, NC, USA).

## Results

### Patients

A total of 124 patients with locally incurable R/M HNSCC treated with first line platinum-based chemotherapy in association with cetuximab were included in this retrospective study. The baseline clinical/pathological features are reported in Table 1.

**Table 1:** Clinical features

Parameter	EXTREME	PCC schedule	Total	p value
N	103	21	124	
Age, mean (SD)	68.2 (11.1)	69.3 (10.8)	68.4 (11.0)	0.6798
< 65	33 (32.0)	6 (28.6)	39 (31.5)	0.947
65–75	41 (39.8)	9 (42.9)	50 (40.3)	
> 75	29 (28.2)	6 (28.6)	35 (28.2)	
Male	72 (69.9)	17 (81.0)	89 (71.8)	0.427
Alcohol	29 (28.2)	4 (19.0)	33 (26.6)	0.588
Smoke during treatment	22 (21.4)	4 (19.0)	26 (21.0)	1.000
Baseline PS				
0	39 (37.9)	0 (0)	39 (31.5)	< 0.0001
1	64 (52.4)	0 (0)	64 (52.4)	
2	0	21 (100)	21(26.04)	
Toxicity all G	70 (68.0)	11 (53.0)	70 (56.5)	< 0.0001

89 (72%) patients were male and median age was 68.4 (SD 11) years. Baseline ECOG PS, evaluated before the start of chemotherapy, was 0, 1, and 2 in 39 (32%), 64 (52%) and 21 (17%) patients, respectively. The primary tumor site was the oropharynx in 18 patients (15%), the hypopharynx in 8 patients (6%), the larynx in 40 patients (32%), the oral cavity in 50 patients (40%), and the paranasal sinus in 8 patients (7%). Histological type was squamous cell carcinoma in all the patients. Human papilloma virus (HPV) status was positive in 2/20 oropharyngeal cancer patients.

Continued smoking during treatment as well as previous alcohol abuse were reported in 26 (21%) and 33 (27%) of patients, respectively. The site of relapse was locoregional, metastatic or both in 50 (40%), 59 (48%) and 15 (12%) of patients, respectively. 103 patients (83%) received standard first line platinum-based chemotherapy, according to the phase III EXTREME trial, while 21 patients (17%) received first line PCC weekly chemotherapy.

As shown in Table 1, no significant difference was reported in terms of age, sex, smoking and previous alcohol abuse between the two distinct subgroups of patients based on the received chemotherapy schedule. On the other hand, patients in the EXTREME group were ECOG PS 0 (39) and 1 (64) while patients (21) in the PCC scheme group were all PS 2.

70 patients (68%) in the EXTREME group experienced chemotherapy related toxicity. High grade (G3) neutropenia, anemia and gastrointestinal toxicity was reported in 23 (22%), 13 (13%) and 9 patients (9%) respectively, in accordance with the expected strong toxicity profile.

In the weekly PCC group 11 patients (52%) reported G1 anemia, 8 patients (40%) reported G2 neutropenia and 10 patients (49%) reported G1 gastrointestinal toxicity. No G3-4 toxicities were reported.

### Outcomes

**Fig. 1:**
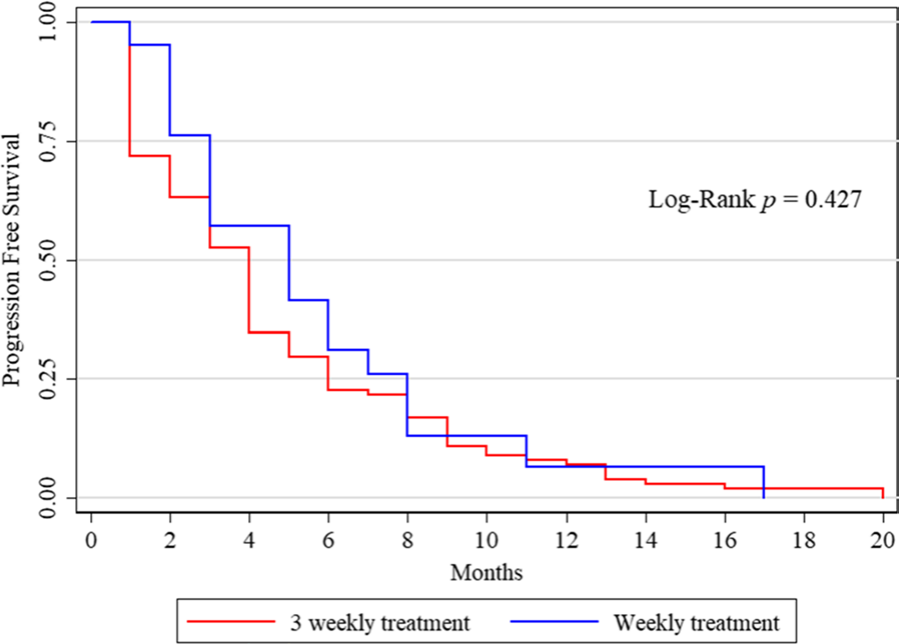
Kaplan Meyer curves. No statistically significant difference between the EXTREME and PCC schedule subgroups was reported in terms of PFS (p value 0.427)

PFS was 4 months (range 1–20) in the overall population; median PFS was 4 and 5 months in the EXTREME and PCC schedule groups, respectively. No statistically significant difference was found in PFS between the two treatment groups (p = 0.275, Fig. 1). On the univariate analysis (Table 2), no clinical features, including the type of treatment regimen, were associated with the PFS.

**Table 2:** Univariate Cox analysis: association between clinical characteristics and PFS

Parameter	HR	95% HR CI		p value
Gender (m vs f)	0.98	0.66	1.47	0.935
Age				
< 65	1.00	1.00	1.00	
65–75	0.87	0.57	1.34	0.526
> 75	1.06	0.67	1.69	0.797
Alcohol	1.16	0.77	1.73	0.484
Smoke	0.95	0.61	1.48	0.804
Baseline PS				
0	1.00	1.00	1.00	
1	1.12	0.74	1.69	0.598
2	1.00	0.62	1.62	0.992
Toxicity	0.77	0.53	1.12	0.169
PTC vs Extreme)	0.48	0.84	0.51	0.505

**Fig. 2:**
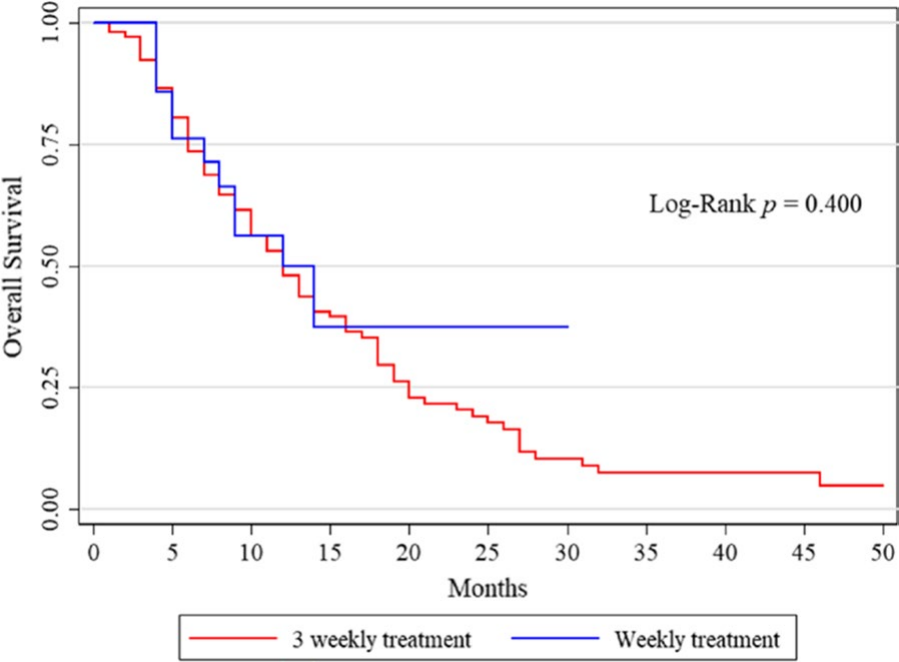
Kaplan Meyer curves. No statistically significant differences between the EXTREME and PTC schedule subgroups were reported, in terms of OS in our patient population (p value 0.400)

OS was 12 months (range 1–50) in the overall population; median OS was 12 months both in the EXTREME and PCC schedule groups. No statistically significant difference was found in OS between the two treatment subgroups (p = 0.400, Fig. 2). On the univariate analysis (Table 3), none of the clinical features, including the type of treatment regimen, were found to be predictive of survival.

**Table 3:** Univariate Cox analysis: association between clinical characteristics and OS

Parameter	HR	95% HR CI		p value
Gender (m vs f)	1.09	0.70	1.70	0.690
Age				
< 65	1.00	1.00	1.00	
65–75	0.77	0.47	1.24	0.282
> 75	1.03	0.63	1.70	0.894
Alcohol	1.30	0.84	2.02	0.244
Smoke	0.93	0.57	1.52	0.778
Baseline PS				
0	1.00	1.00	1.00	
1	0.91	0.58	1.42	0.675
2	0.98	0.58	1.68	0.951
Toxicity	1.21	0.79	1.85	0.375
PCC vs. Extreme	0.78	0.42	1.43	0.416

The response rate was 35% in EXTREME group (36 patients) and 34% in PCC group (7 patients) with a relevant palliative effect. None of the patients of both groups achieved a CR.

## Discussion

In this retrospective study frail patients treated with the PCC chemotherapy regimen had similar PFS compared to fit patients treated with the EXTREME schedule (p = 0.275) in a first line setting. These data support the concept of modulating the chemotherapy schedule based on the patient's general condition as a reasonable and safe approach, which does not compromise disease control in frail patients.

In a daily clinical practice setting the coexistence of several factors often makes patients with locally incurable R/M HNSCC fragile and difficult to manage: they often have an ECOG PS > 1, advanced age, severe symptoms and relevant comorbidities (such as diabetes mellitus type 2, arterial hypertension and cardiopathies) which often limit the use of chemotherapy. Moreover, patients with R/M HNSCC are characterized by frequent malnutrition and poor habits and social status [14]. Consequently, there is an urgent need to customize the therapeutic approach in order to decrease toxicities and morbidities without compromising the oncological outcomes.

In contrast to the PS 0–1 populations often included in randomized clinical trials, the EXTREME regimen is contraindicated in PS 2 patients, who often make up the majority of the patients seen in real-world clinical practice for the high risk of infectious diseases and high-grade toxicities. Therefore, cetuximab was evaluated in combination with different platinum-based regimens [15, 16]. Several retrospective and prospective studies have examined first-line chemotherapy based on a weekly combination of paclitaxel and cetuximab in R/M-HNSCCs, showing promising activity [11, 17, 18]. This schedule has proven to be a relevant therapeutic option for patients with poor prognosis considered unsuitable for the EXTREME regimen [17].

In a retrospective study the combination of paclitaxel and cetuximab significantly prolonged the PFS compared to the EXTREME regimen, mostly in older male patients and in patients with tracheostomy [18].

Pêtre et al. evaluated the activity and safety of the weekly carboplatin and paclitaxel chemotherapy in patients with R/M HNSCC unfit for cisplatin due to comorbidities and poor baseline clinical condition. Weekly chemotherapy was efficient and well tolerated in this subgroup of particularly fragile patients [11].

In this study the PCC chemotherapy regimen, used in a population with poor baseline clinical conditions showed a non-inferior efficacy profile, with an excellent tolerability compared to the EXTREME regimen administered in PS 0–1 fit patients. Furthermore, the safety profile was optimal with no high-grade toxicity, compared to the heavy toxicity profile reported in the EXTREME group.

The efficacy is probably due to this optimal tolerability profile, demonstrated by the low degree of toxicity, which allowed an optimal dose intensity even in this group of frail patients. Tolerability could be correlated to the flexibility of the weekly treatment which allows to delay and/or reduce the dose as needed. Our data are in line with the ones from Naverson et al. who have proved that the use of PCC in patients affected by R/M-HNSCC with PS 0–2 appears to be a profitable therapeutic strategy with low toxicity [19]. This retrospective analysis highlighted that 41% of patients showed PR or CR and 34% of patients showed SD. Similarly, in our experience the PCC chemotherapy regimen obtained 34% of ORR, demonstrating a relevant cytoreductive effect compared to the EXTREME one.

Recently, the phase III Keynote 048 study led to the approval of pembrolizumab, either alone or in combination with platinum/5FU chemotherapy in first line in R/M HNSCCs expressing PD-L1, showing an advantage in terms of OS when compared to standard EXTREME regimen in patients with tumours expressing a combined proportion score (CPS) of 1 or higher ^13.^ Pembrolizumab in monotherapy could be the first choice in frail patients, since they will not be able to receive chemo/immunotherapy due to the high toxicity rate of the combination. However, the Keynote 048 study included a population of PS 0–1 patients with no comorbidities. Therefore, patients with poor clinical conditions and comorbidities were not represented in this clinical trial and unfortunately there is no data regarding the association of pembrolizumab with weekly platinum-based chemotherapy schedule.

**Fig. 3:**
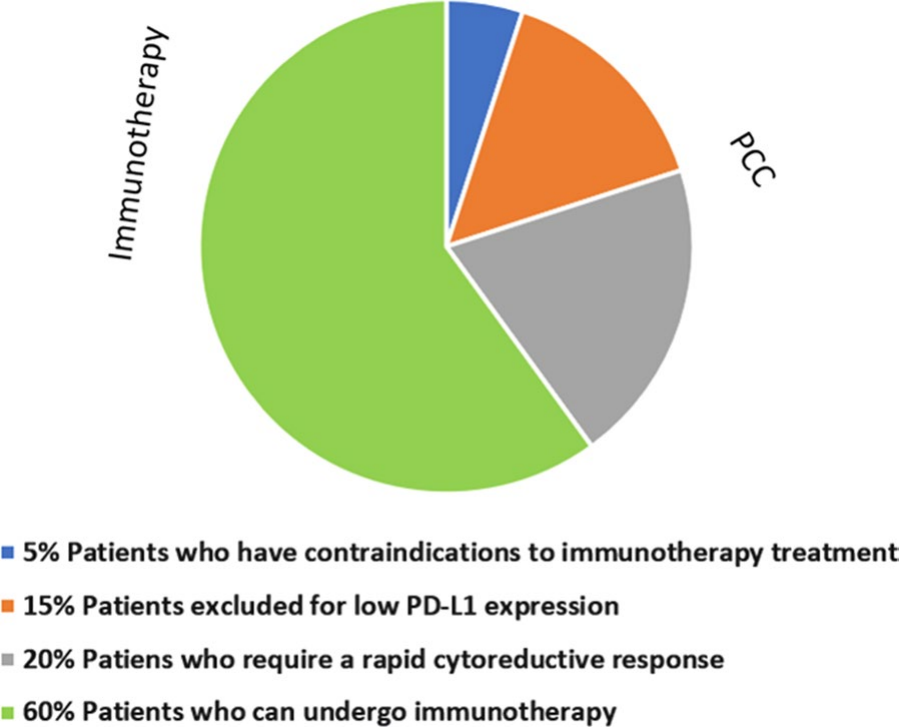
First line in frail patients with R/M HNSCC. Weekly chemotherapy treatment is placed in the group of patients excluded from immunotherapy treatment for low PD-L1 expression (15%), for contraindications to immunotherapy treatment for uncontrolled infectious or autoimmune diseases or organ transplantation (5%) or requiring a rapid cytoreductive response (20%)

The PCC schedule could be considered as a first line treatment in all frail patients excluded from pembrolizumab treatment, for its optimal efficacy and toxicity profile or in relation to CPS < 1 or due to the presence of clinical contraindications to immunotherapy treatment such as in autoimmune diseases or uncontrolled infections. A third group of patients which could potentially be a candidate for the PCC treatment is represented by those with frail condition requiring a rapid tumour response, as HNSCC is an aggressive disease often associated to rapid worsening symptoms, such as pain, breathing and feeding difficulties (Fig. 3). In these first and third fragile patient groups immunotherapy with the anti-PD-1 nivolumab can be considered in second-line platinum-resistant disease, allowing for a rational therapeutic sequence [20].

This study has some limitations due to its monocentric and retrospective nature. On the other hand, its strength lies on the evaluation of a frail population which is usually excluded from randomized clinical trials. The challenge is to define a customized first-line approach in head and neck cancer patients without excluding frail patients, considering patient preferences, socio-economic status and the availability of caregivers. Each treatment should be determined considering PS and comorbidity, symptoms and CPS, the need of rapid response, risk of complications and site of relapse [21], necessarily in the context of a multidisciplinary team evaluation. This tailored approach could lead to excellent oncological outcomes, gaining disease control without compromising the patient's quality of life.

In conclusion, a PCC schedule appears indicated in frail patients and could currently be considered in all frail patients excluded from immunotherapy treatment (CPS < 1) or when immunotherapy treatment has contraindicated as well as when a prompt and strong response is required for disease control in critical sites. It also appears advisable to explore the combination of check point inhibitors with modulated chemotherapy regimens in frail patients in the near future.

Weekly chemotherapy treatment is placed in the group of patients excluded from immunotherapy treatment for low PD-L1 expression (15%), for contraindications to immunotherapy treatment for uncontrolled infectious or autoimmune diseases or organ transplantation (5%) or requiring a rapid cytoreductive response (20%).

## Data Availability

All data generated or analysed during this study are included in this published article [and its supplementary information files].
